# Supplementary material for: What Is Accounting for the Rapid Decline in Cigarette Sales in Japan?
Source: Int J Environ Res Public Health. 2020 May 20;17(10):3570. doi: 10.3390/ijerph17103570 (PMC7277739; doi:10.3390/ijerph17103570)

Supplementary Figure 1. Joinplot analysis of tobacco products (billion sticks). * indicates that the slope is significantly different from zero, alpha = 0.05.

Total (cigarettes + HTPs), 2011-2019

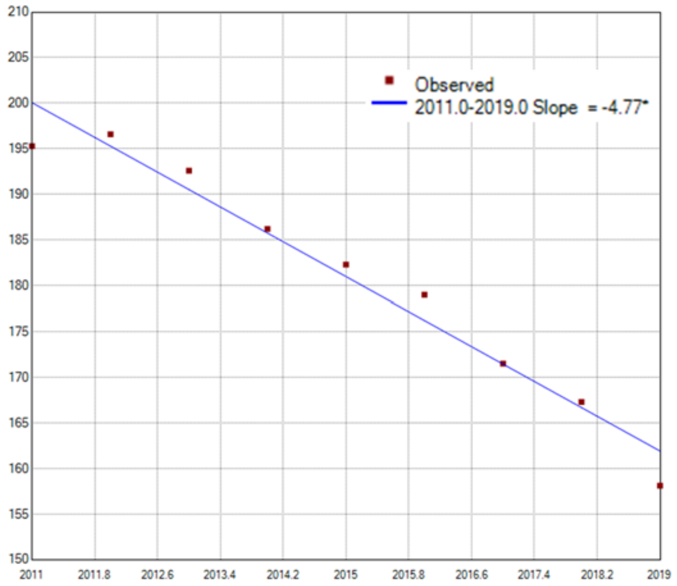

Cigarettes only, 2011-2019

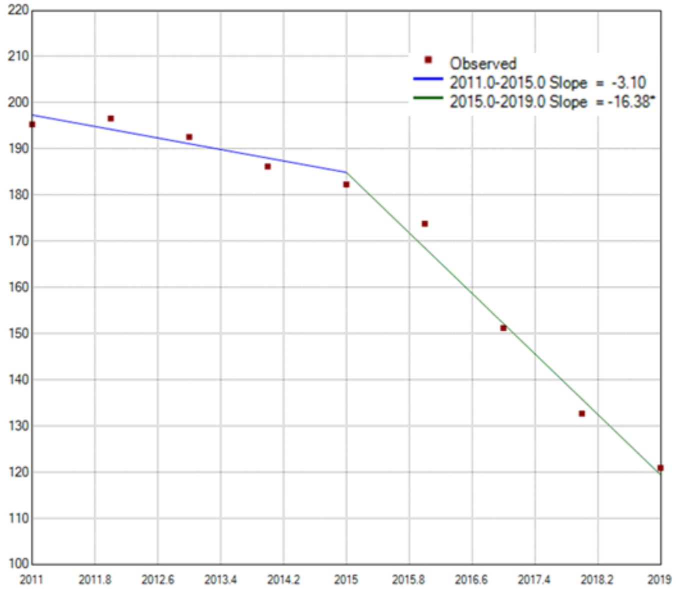

Total (cigarettes + HTPs), 2016-2019

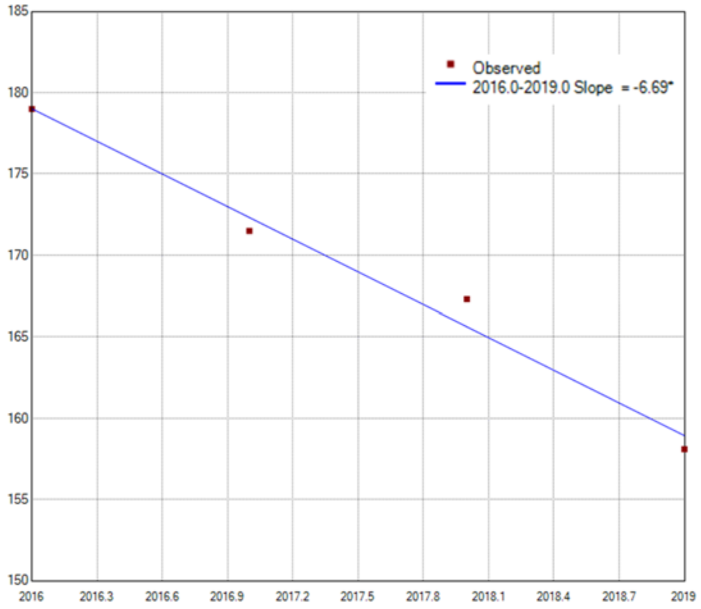

Cigarettes only, 2016-2019

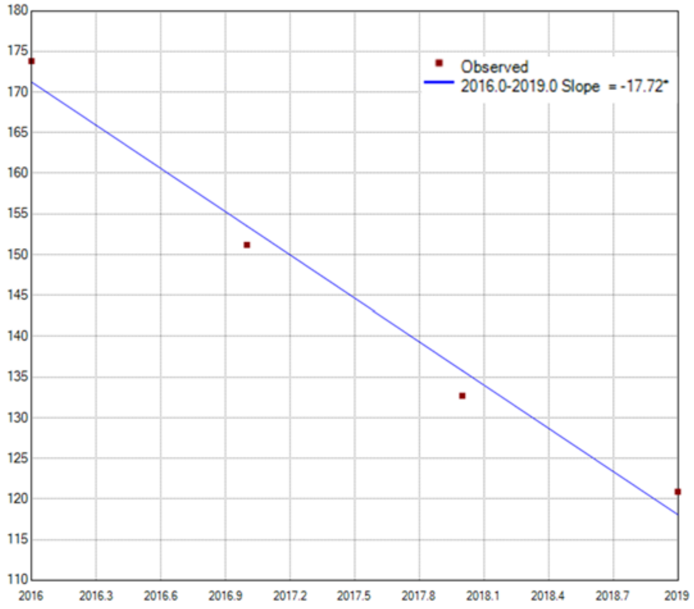

Supplement: Supplementary file 1 [file ijerph-17-03570-s001.pdf]
